# Accelerometric assessment of fatigue-induced changes in swimming technique in high performance adolescent athletes

**DOI:** 10.1038/s41598-024-83310-w

**Published:** 2025-01-18

**Authors:** Maciej Skorulski, Małgorzata Stachowicz, Szymon Kuliś, Jan Gajewski

**Affiliations:** Józef Piłsudski University of Physical Education on Warsaw, Warsaw, Poland

**Keywords:** Skeletal muscle, Fatigue

## Abstract

The present study analyzed the kinematic changes under fatigue in highly trained adolescent swimmers during a 50-m all-out front cwal test. Twenty-four girls and fourteen boys aged 12–13 participated in the study. The movement of the hip rim was analyzed using a specialized inertial device equipped with a triaxial gyroscope and accelerometer to measure changes in angular velocity and acceleration. Between the first and second lengths of the pool, the following were observed: a significant (F1.36 = 63.6; *p* < 0.0001; η^2^ = 0.64) increase (34%) in maximum pelvic angle, significant (F1,36 = 6.0; *p* = 0.0193; η^2^ = 0.14;) increase (12.10%) in angular velocity in rotational motion around a vertical axis, and a significant (F1,36 = 11.29; *p* = 0.0018; η^2^ = 0.24) increase (6.86%) in angular velocity in yaw rotation motion around the sagittal axis. Significant (F1,36 = 13.96; *p* = 0.0006; η^2^ = 0.28) differences in maximum pelvic angle were observed for lap and side. As unfavourable changes in kinematics are already observed in the second half of the distance, it is therefore suspected that performing frequent high-intensity repetitions may lead to the perpetuation of unfavourable movement patterns. Taking this into account, coaches should limit maximum-speed swimming in adolescent athletes to short distances and an appropriate interval and use training methods to reduce asymmetric work such as training snorkels.

## Introduction

Swimming is a sport in which swimming technique has a significant impact on athletic performance^1^, and the development of movement technique and improvement of strength are two major issues in swimming biomechanics^2^. The literature indicates that swimming performance is highly dependent on variables related to technical parameters^3^. In athletes of younger age (12–14 years), swimming training should focus particularly on improving technique^4^, due to the increased susceptibility of adolescent swimmers to technical changes^5^.

In recent years, biomechanical models determining swimming sports performance have been determined using: a three-dimensional (3D) analysis method^6^, a swim speedometer method^7^, and an accelerometer method^8^. By using the 3D method, it is possible to analyze not only intra-cyclic velocity variations, but also the displacement and acceleration of the body’s center of mass^9,10^ and angular velocity of the upper limbs in swimmers^11^. Unfortunately, this method is very time consuming and depends on the accuracy of the biomechanical model^12^. Because of this, variability between cycles is excluded, limiting the method’s utility for practical training purposes.

In contrast, the swim speedometer method, a cord attached to the swimmer’s waist belt is used to calculate changes in the intra-cyclic velocity at which the athlete moves. This method is limited by taking measurements in only one part of the swimming pool^13^. The use of an accelerometer and gyroscope attached to a particular body segment allows for analysis of many swim cycles, fatigue evaluation, and quick feedback for coaches and athletes^8^. This type of measurement is definitely easier to perform and cheaper than most of the methods described. With the right software, it is possible to obtain very accurate information about the movement kinematics of the players^14,15^. All these analyses and research devices enable further precise research, contributing to a closer understanding of issues related to swimming technique and the forces acting on the swimmer’s body, as well as the adaptation of the athlete’s body to training loads.

Research results reported by Aujouannet et al.^16^ indicate that swimming speed depends on distance per stroke (DPS), stroke rate (SR) and intracycle velocity variation (IVV). IVV is a biomechanical variable that describes the variation in velocity during the swim cycle. IVV reflects the interaction between propulsive and resistive forces^5^. It has been observed that greater fluctuations in these parameters during races occur in lower-performance athletes^17^. High-performance athletes are able to reduce their effort in the preparatory phase in favor of the active phase under fatigue conditions, maintaining the continuity of propulsion^18^.

Swimming speed also depends on body rotation along the vertical axis (roll rotation). During this roll motion, the swimmer can reduce frontal drag in freestyle and backstroke, and thus cover the distance with less effort^19^. In addition, the streamlined position of the body in freestyle creates opportunities to adopt a convenient position for inhalation without too much involvement of the muscles taking part in respiratory activities^20^.

Chollet et al.^21^ proposed an index of coordination for freestyle (IdC), which determines the delay that occurs between the propulsion of one and the other upper limb. Based on subsequent studies, the IdC was found to be positively correlated with swimming speed and energy expenditure^17^, because as swimming speed increases, active resistance also increases^22^. Ana Silva et al.^23^ pointed out that the IdC may depend on individual physiological and biomechanical responses related to fatigue, where swimming technique will have an important role.

Fluctuations in intra-cyclic velocity during the swim cycle are the result of acceleration and deceleration occurring due to the propulsive movements of the upper and lower limbs and the resistance that the water environment exerts on the swimmer’s body. In front crawl, there are three main phases of arm movement under the water surface: downsweep, insweep, and upsweep, which form the basis of upper limb propulsion. The swimmer can make more efficient use of upper limb propulsion by reducing the velocity fluctuations between the propulsive phases of the arms, thus achieving lower IVV, reduced active drug, and energy cost of effort^24–26^.

Adolescent swimmers have rarely been measured with accelerometers^5^ despite the fact that the development of the technique at this period of the training process is crucial. Technical changes in adolescent athletes can occur under the influence of rapid development but also inappropriate training loads. Therefore, it is reasonable to regularly monitor kinematic changes in young swimmers, which provide objective, precise data that can be compared and analyzed^7,13^.

We know from research that under the influence of fatigue there are many adverse kinematic changes in athletes^27^. The aim of the study was to evaluate the technical changes occurring after 25 m of swimming at maximum velocity in the crawl in adolescent swimmers. These changes can impact both the efficiency of the swimming stroke and the overall performance. Understanding these alterations is crucial for developing more effective training strategies to mitigate the negative effects of fatigue.

Kinematic changes, such as loss of efficiency in arm movement, body rotation, or postural stability, can lead to reduced performance, which is particularly evident over short distances^26^. We know from research that under the influence of fatigue, there are many adverse technical kinematic changes in athletes^27^. There are few accelerometer studies on swimming kinematics in children and adolescents under fatigue conditions. Such studies have mainly focused on describing simple variables such as stride length and frequency. Thus, the aim of this study was to identify patterns of fatigue-related kinematics changes in young athletes during short distance maximal speed front crawl swimming.

## Materials and methods

### Participants

The study included 24 girls (age 12.57 ± 0.55 years; body height 164.1 ± 8.3 cm; body mass 51.3 ± 8.4 kg; body fat 20.2% ± 2.9%; 50 m crawl swim time 32.15 ± 2 s; FINA points 453.5 ± 83.36 ) and 14 boys (age 12.51 ± 0.6 years; body height 166 ± 8.3 cm; body mass 49.1 ± 9.4 kg; body fat 13.6% ± 2.0%; 50 m crawl swim time 30.4 ± 2.8 s, FINA points 391.0 ± 60.2). All of the subjects were participants in a Youth National Team training camp in the Mazovia Province, to which athletes are appointed who rank in the top ten in their age category at Olympic distances in Poland based on www.swimrankings.net.

The study was approved by the Senate Committee on Research Ethics of the Józef Piłsudski Academy of Physical Education in Warsaw (SKE 01–31/2023). All procedures involving human participants were performed in accordance with relevant guidelines and regulations, and in adherence to the Declaration of Helsinki. Informed consent was obtained from all participants and, due to the age of the subjects, additionally informed consent was obtained from their parents or legal guardians. Participants and their legal guardians were fully informed about the nature, purpose, and course of the study, including any potential risks or benefits associated with participation.

Measurements were taken in a 25-meter swimming pool. The subjects’ task was to swim a distance of 50 m at maximum speed. Instead of jumping from the starting blocks, the athletes began by pushing off from the pool wall. The athletes swam two laps of the swimming pool along the way making a flip turn typical of front crawl. Before the measurement, the athletes performed a warm-up consisted of 1200 m^28^ and was organized into 3 parts. I part aimed at reducing muscle stiffness, risk of injury by warming up a large number of muscles^29^. II part to stimulate aerobic pathways, increase blood flow in the movements of which the main task will be performed (after the warm-up)^30^. III part stimulation of the nervous, swimming with restricted breathing and muscular systems preparation for maximum performance^31^.

- Warmup:
- 300 m free to choose.
- 12 × 25 m medley start in 40 s.
- 300 m crawl (very easy)
- 3 × 100 m start in 120 Sect. (1 × 10 m crawl all-out / 90 m very easy; 1x progressive with increasing speed; 1x breathing every 5 strokes)

Measurements were taken during the same training period, according to a common calendar of major events for athletes aged 12–13.

### Instruments

**Fig. 1:**
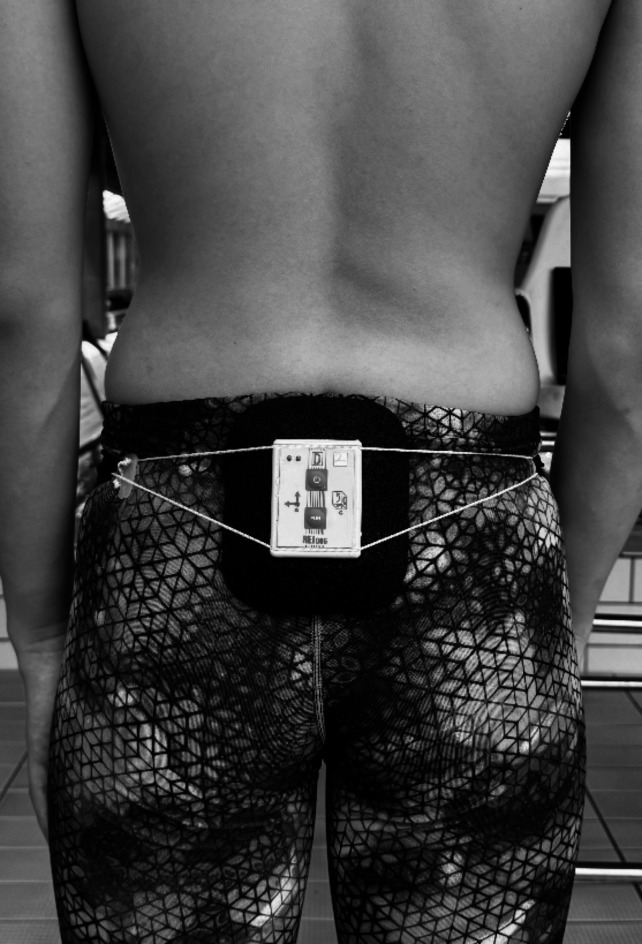
Method of fixing of the device for direct recording of triaxial accelerations and angular velocities of rotation.

For each athlete, the individual characteristics of the kinematics of the hip girdle movement during the averaged cycle for the first and second lap were calculated. A special inertial device with a built-in triaxial gyroscope and triaxial accelerometer was used to measure and record changes in velocity and acceleration (REJ62g, JD Jarosław Doliński, Poland). The device (65 × 50 × 30 mm, 95 g) was placed in the foam in such a way as to minimize hydrodynamic drag while ensuring stable restraint on the dorsal aspect of the swimmer’s iliac rim. The center of the recorder was located at the level of the base of the sacrum. The recorder was attached using a special belt consisting of two parts: an inextensible rope to which the recorder was attached and an elastic band placed on the subject’s lower abdomen (Fig. 1).

Measured signals were sampled at 200 Hz. A measurement range of ± 2 *g* (1 g = 9.81 m·s^−2^) was used to measure accelerations. The signal was subjected to low pass filtering with a cutoff frequency of 93 Hz. To measure the angular velocity of rotation, a measuring range of ± 500 deg.s^−1^ was used.

According to the manufacturer, absolute error of acceleration measurement was ± 0.2 m.s^−2^. The accuracy of rotation angular velocity measurements was checked indirectly by measurement and calculation of the angle of rotation of the recorder in the range of 90 degrees around each axis. The absolute error of angle calculations was ± 1 deg. The absolute error of measurements of the speed of angular rotation was estimated at 0.6 deg.s^−1^.

The measured waveforms were smoothed with a low-pass, four-pole Butterworth filter with a cutoff frequency of 20 Hz. The filter cut-off frequency was chosen using the assumption that, as a result of filtering the measured signals, the calculated amplitude of changes in the speed of progressive motion would not be attenuated by more than 0.5%.

The waveforms of the acceleration and angular velocity components over the individual cycles were averaged for each lap. The waveforms of the standard deviation of the analyzed variables were also calculated. The calculations were carried out utilizing the author’s software, STA1v0, developed by Zbigniew Staniak at the Institute of Sport – National Research Institute in Poland.

The following variables calculated for averaged cycle were analyzed: av_max_ – maximal acceleration along the vertical axis in translational motion, ω_max_R – maximal angular velocity around the vertical axis in rotational movements (roll), ω_max_Y – maximal angular velocity around the sagittal axis in yaw movements (yaw rotation) and A_max_R – the maximum angle of pelvic tilt about the vertical axis in rotational movements (Fig. 2). The analysis considered the values separately for movements during activity of the left upper limb (Left) and right upper limb (Right), and separately for the first lap (I25) and the second lap (II25).

The Tanita BC-545 N balance (Tanita Corporation, Tokyo, Japan) was used to measure weight and body composition.

### Procedures

**Fig. 2:**
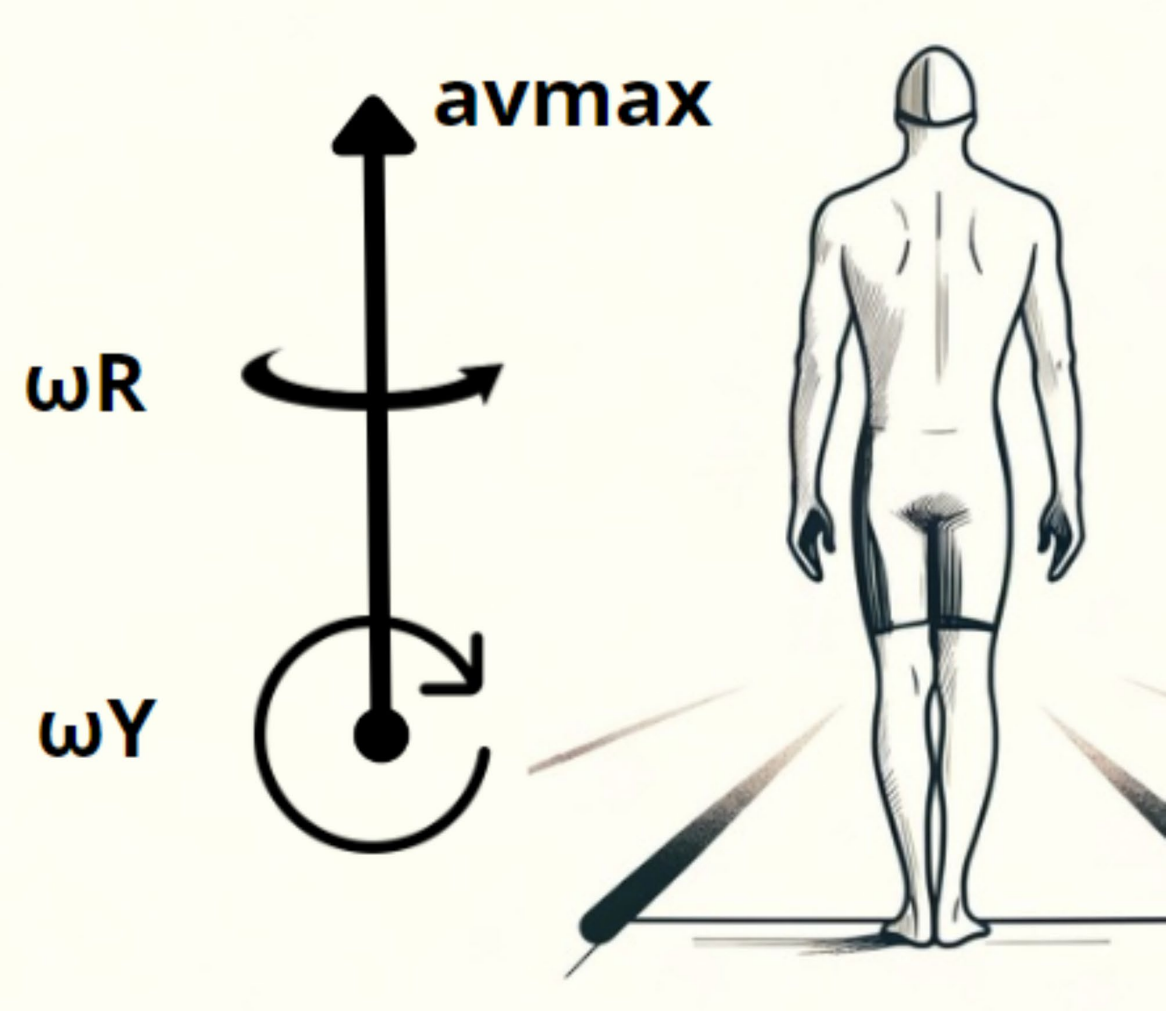
layout and orientation of the measurement axes of triaxial accelerations and angular velocities of rotation. Notes: acceleration components: Notes: acceleration components: avmax – along the vertical axis and components of angular velocities of rotation: ωR – around the vertical axis, ωY - around the sagittal axis of swimmer’s body.

### Statistical analysis

Using the G*POWER program, it was calculated that 34 people (17 + 17) would be needed. The assumption was made to detect an interaction with a mean effect size at a significance level of 0.05 and a statistical power of 0.80).

Statistical analyses were performed using STATISTICA v. 13.1 software (TIBCO Software Inc., 2017). The normality of the data distribution was assessed using the Kolmogorov-Smirnov test, with a p-value threshold of > 0.20 used to confirm normality of a distribution. None of the analyzed variables deviated from normality.

To examine differences between means, a repeated measures ANOVA (general linear model) was applied. The analysis considered two repeated factors: LAP (first and second) and SIDE (left and right), while GENDER (female, male) was included as a fixed factor. For post-hoc comparisons, the Tukey test was used. The significance level was set at α = 0.05. Results were presented as means ± standard deviations, as well as 95% confidence intervals. Effect sizes were calculated using partial eta squared. Results were presented as means +/- and standard deviations, as well as 95% confidence intervals.

## Results

The mean ± SD, p, F, and η^2^ values for the variables analyzed are shown in Table 1. Significant changes in kinematic parameters were observed in the II25 and the difference in swimming technique during the right and left hand strokes, as evidenced by the observed significance and effect sizes of the analyzed variables.

**Table 1:** Mean ± SD, p-values, F and η^2^ for the analyzed components of acceleration for the first and second lengths of the pool.

Variables	Side	I25	II25	Side effect	Part effect	Side x Part
av_max_ (m*s^–2^)	Left	8.6 ± 1.8 (8.03–9.17)	8.1 ± 1.9 (7.50–8.70)	F_1,36_= 17.94; *p* < 0.0001; η^2^ = 0.33	F_1,36_= 16.02; *p* = 0.0003; η^2^ = 0.31	F_1,36_= 0.55; *p* = 0.465; η2 = 0.015
	Right	7.7 ± 1.8 (7.1–8.3)	6.8 ± 1.6 (6.3–7.3)			
ω_max_Y (deg/s)	Left	102.5 ± 23.9 (94.9–110.1)	110.0 ± 22.4 (102.9–117.1)	F_1,36_= 1.72; *p* = 0.1973; η2 = 0.04	F_1,36_= 11.29; *p* = 0.0018; η^2^ = 0.24	F_1,36_= 1.0; *p* = 0.3126; η2 = 0.03
	Right	100.1 ± 22.0 (93.1–107.1)	106.4 ± 24.7 (98.5–114.3)			
t_ω_max_Y (s)	Left	0.31 ± 0.08 (0.28–0.34)	0.41 ± 0.16 0.36–0.46	F_1,36_= 0.001; *p* = 0.97; η2 < 0.001	F_1,36_= 36.2; *p* < 0.0001; η^2^ = 0.50	F_1,36_= 0.22; *p* = 0.6414; η2 = 0.006
	Right	0.32 ± 0.10 (0.29–0.35)	0.41 ± 0.12 (0.37–0.45)			
A_max_R (deg)	Left	29.1 ± 6.3 (27.10–31.10)	42.3 ± 7.1*** ^^^ (40.04–44.56)	F_1,36_= 13.7; *p* = 0.0007; η^2^ = 0.27	F_1.36_= 63.6; *p* < 0.0001; η^2^ = 0.64	F_1,36_= 13.96; *p* = 0.0006; η^2^ = 0.28
	Right	29.6 ± 6.1 (27.66–31.54)	36.9 ± 6.1** (34.96–38.84)			
t_A_max_R (s)	Left	0.55 ± 0.09 (0.52–0.58)	0.73 ± 0.24 (0.65–0.81)	F_1,36_= 4.78 ; *p* = 0.0353; η^2^ = 0.12	F_1,36_= 39.1; *p* < 0.0001; η^2^ = 0.52	F_1,36_= 2.4; *p* = 0.1326; η^2^ = 0.06
	Right	0.52 ± 0.07 (0.50–0.54)	0.65 ± 0.14 (0.61–0.69)			
ω_max_R (deg/s)	Left	246.2 ± 51.7 (229.8–262.6)	274.9 ± 52.7 (258.1–291.7)	F_1,36_= 0.2; *p* = 0.6433; η^2^ = 0.006;	F_1,36_= 6.0; *p* = 0.0193; η^2^ = 0.14;	F_1,36_= 2.7; *p* = 0.1402; η^2^ = 0.07
	Right	237.9 ± 58.9 (219.2–256.6)	265.7 ± 45.6 (251.2–280.2)			
t_ω_max_R (s)	Left	0.31 ± 0.10 (0.28–0.34)	0.50 ± 0.22*** ^^^ (0.43–0.57)	F_1,36_=12.16; *p* = 0.0013; η^2^ = 0.25	F_1,36_= 23.16; *p* < 0.0001; η^2^ = 0.39	F_1,36_= 7.16; *p* = 0.0111; η^2^ = 0.17
	Right	0.28 ± 0.11 (0.25–0.31)	0.38 ± 0.17*** (0.33–0.43)			

Left – motion during active left upper limb, Right – motion during active right upper limb, I25 – the first part (25 m) of the analyzed effort, II25 – the second part (25 m) of the analyzed effort, Side effect – statistical interaction between Left and Right, Part effect – statistical interaction between I25 and II25, av_max_ – acceleration along the vertical axis in traversing movements, ω_max_Y – maximum angular velocity around the sagittal axis of the athletes in yaw movements (yaw rotation), t_ω_max_Y – time needed to achieve maximum angular velocity around the sagittal axis of the athletes in yaw movements (yaw rotation), AmaxR – maximum angle around the long axis in rotation movements, t_A_max_R – time needed to achieve maximum angle around the long axis in rotation movements, ω_max_R – maximum angular velocity about the long axis in rotation movements, t_ω_max_R – time needed to achieve maximum angular velocity around the long axis (roll). Different than I25: *< 0.05, **< 0.01, ***< 0.001. Different than Right: ^< 0.05, ^^< 0.01, ^^^< 0.001.

The maximum value of the av_max_ significantly decreased by 8.5% on the II25, and significant differences in acceleration values were also observed when creating propulsion with the right and left hands.

The analysis showed a significant interaction between LAP and GENDER (F_1,36_ = 32.0, *p* < 0.005, η^2^ = 0.31), which was associated with a decrease in acceleration values in the second lap that was greater for boys than girls.

Values of the ω_max_Y in the tested athletes increased significantly by 6.86% in the II25. The same effect was also observed in the time required to reach the ω_max_Y (t_ω_max_Y). The amount of change between I25 and II25 was 32%.

LAP and SIDE effects were observed in the value of the A_max_R. On the II25, the value of A_max_R increased by 34%. The difference between the left and right strokes at the I25 was 1.95%, and at II25 this difference was 12.76%. An interaction was observed between LAP and SIDE. The post-hoc test performed showed significant differences between the I25 and II25 during the propulsive movements of the left and right arms, as well as a difference during II25 between the left and right arm propulsive movements.

Time required to achieve A_max_R (t_A_max_R) increased on the II25 by 33.01%. For the same value, a significant difference was observed between the left and right, which was 5.45% on the I25 and 10.95% on the II25. However, no interaction was observed between the part of the distance swum (LAP) and the active phase of the left or right arm (SIDE).

A significant small magnitude increase of 12.10% in the value of the ω_max_R was observed between I25 and II25.

On the other hand, the time required to achieve ω_max_R (t_ω_max_R) increased on the II25 by 51.72% compared to the I25. The observed increase was significant, with a large effect size. Above that, significant differences of average effect were observed between the movements during the left and right arms. The study also showed an interaction between LAP and SIDE. Post-hoc analysis showed significant differences in body movement during left arm and right arm work between the I25 and II25, and between left and right arm action on the II25. No significant differences were observed in the movement of the right and left hands on the I25.

## Discussion

The study presents an analysis ofthe kinematic changes occurring under fatigue in athletes. The measurement method used proved to be an easy, detailed, and fast measurement tool for assessing swimming kinematic in adolescent swimmers. It can be used to analyze fatigue in athletes and the kinematic changes that go with it over more than one length of the swimming poll.

Accelerometer measurements showed a number of significant changes in the rotational movements of the body around the vertical axis in the II25 during maximum speed crawl swimming in high-performance adolescent swimmers. A pronounced increase in A_max_R coincides with the results of the study by Psycharakis and Sanders^10^, who observed increased hip rotation under fatigue in swimmers. Because increasing rotation makes it easier to take a breath^32^, this may explain the asymmetry in the maximum angle inclination of the pelvis at II25, where athletes under fatigue take more breaths, and we know from research that unilateral breathing is common in athletes^33^.

The differences observed in AmaxR and t_ AmaxR during the active phase of the right and left arms on II25 indicate a lack of symmetrical operation. Asymmetries were also observed by a significant difference in Avmax during the propulsive phase of the left and right arms. Previous studies^29,30^ proved that the fastest swimmers were characterized by a lower asymmetry of propulsive forces and that the asymmetry was not associated with a preference for the respiratory side.

Asymmetry is one of the key aspects between fatigue and the changes we observed in this study. Above that, Seifert et al.^34^emphasized that coordination management in kraul is particularly important in competitive athletes, where these subtle differences induce increased resistance and energy expenditure. This suggests that fatigue significantly interferes with coordination which may hinder future performance of athletes. Optimizing asymmetry in swim training is a direction that can improve performance. A study by Vullings et al.^35^ shows that using an accelerometer to monitor athletes in real time provides enough information to correct technique before fatigue-induced impairments take root.

Theoretically, maintaining symmetrical work and applying similar propulsive forces when working with the left and right upper limbs can positively reduce IVV^10,31^ and improve body position in the water, which will directly contribute to improved swimming performance (increased velocity)^37^. As studies have shown, increased body rotation is negatively correlated with speed, which is mainly associated with decreasing strokes frequency^38^ and asymmetric work impairs performance in cyclic and continuous activities such as swimming^39^.

Repetition of movements with incorrect technique is one of the most likely causes of increasing asymmetry^40^. Therefore, coaches should use methods that aim to reduce it in adolescent athletes because during this period there is an increased vulnerability of swimmers to technical changes^5^. Such methods are swimming with bilateral breathing and a training snorkel^32^. Given that adult athletes swim a minimum of 40% of the total training volume with their personal best style^41^, it is necessary to consolidate correct swimming technique before this period in order to make effective use of specialized training.

Therefore, training programs that emphasize bilateral breathing and symmetrical swim cycle mechanics during crawl swimming, especially during high-intensity intervals, could be crucial for the long-term development of adolescent swimmers. Trunk movements around the sagittal axis (yaw rotation), to our knowledge, have been described in the literature in freestyle for the upper body only^35,36^. The results presented in this study suggest an adverse effect on swimming performance because the maximum velocity of yaw movements intensified during II25. The yawing motion of the iliac rim itself logically seems to be unfavorable in sports that rely on translational motion, and in swimming, additionally, such rotational motion will cause increased drag in the propulsive phase.

### Limitations

The limitations of this study should be carefully considered when interpreting the results. Firstly, the study was conducted on a group of athletes aged 12–13 years, and the findings should not be generalized to older athletes. It is possible that adult athletes, particularly those at a higher performance level, experience different kinematic changes under fatigue.

## Conclusion

The primary focus of this study was on assessing kinematic changes over short distances. A goal of every athlete should be to minimize IVV. In addition, especially during short, high-intensity efforts, Additionally, the observed asymmetry that occurs during fatigue is indicative of certain deficits in athletes. Improving such an individual movement pattern can improve athletic performance in athletes.

Repeated high-intensity, high-volume over short distancesin adolescent athletes can lead to the perpetuation of a faulty movement pattern. Taking this into account, coaches should limit maximum-speed swimming in adolescent athletes to short distances and an appropriate interval and use training methods to reduce asymmetric work such as training snorkels. Additionally, they should encourage athletes to perform breathing activity on both sides and pay attention to maintaining symmetrical work despite fatigue or breathing activity.

Future research should aim to address several important areas. One key focus should be the development of training methods that can reduce asymmetry and minimize yaw movements in swimmers. Furthermore, it would be valuable to investigate whether the same correlations between fatigue and kinematic changes are present in older athletes and in different swimming styles as the backstroke. Finally, future studies should assess the extent to which yaw movements, as detected in this research, impact overall sports performance in swimming.

## Data Availability

Data supporting the results of this study have been deposited in RepOD’s open research data repository and are available at the link 10.18150/AL7YXL.
