# Author Correction: Accelerometric assessment of fatigue-induced changes in swimming technique in high performance adolescent athletes

**DOI:** 10.1038/s41598-025-97126-9

**Published:** 2025-04-08

**Authors:** Maciej Skorulski, Małgorzata Stachowicz, Szymon Kuliś, Jan Gajewski

**Affiliations:** Józef Piłsudski University of Physical Education on Warsaw, Warsaw, Poland

Correction to: *Scientific Reports* 10.1038/s41598-024-83310-w, published online 18 January 2025

The Funding section in the original version of this Article was omitted. The Funding section now reads:

“The study was supported by Polish Ministry of Education and Science in the years 2023–2024 under the University Research Project no 3 at Józef Piłsudski University of Physical Education in Warsaw “Postural assessment and accelerometric characterisation of movement technique in selected sports disciplines.”

The original Article has been corrected.

